# Mapping Knowledge Structure and Research Frontiers of Ultrasound-Induced Blood-Brain Barrier Opening: A Scientometric Study

**DOI:** 10.3389/fnins.2021.706105

**Published:** 2021-07-14

**Authors:** Haiyang Wu, Yan Zhou, Lixia Xu, Linjian Tong, Yulin Wang, Baolong Liu, Hua Yan, Zhiming Sun

**Affiliations:** ^1^Clinical College of Neurology, Neurosurgery and Neurorehabilitation, Tianjin Medical University, Tianjin, China; ^2^Tianjin Key Laboratory of Cerebral Vascular and Neurodegenerative Diseases, Tianjin Neurosurgical Institute, Tianjin Huanhu Hospital, Tianjin, China; ^3^Department of Ultrasound, Tianjin Huanhu Hospital, Tianjin, China; ^4^Department of Spine and Spinal Cord, Tianjin Huanhu Hospital, Tianjin, China

**Keywords:** scientometric, knowledge structure, research frontiers, ultrasound, blood-brain barrier, VOS viewer, CiteSpace

## Abstract

**Background:** Among the effective approaches developed for blood-brain barrier (BBB) opening, ultrasound is recognized as a non-invasive technique that can induce localized BBB opening transiently and repeatedly. This technique has aroused broad attention from researchers worldwide, and numerous articles have been published recently. However, no existing study has systematically examined this field from a scientometric perspective. The aim of this study was to summarize the knowledge structure and identify emerging trends and potential hotspots in this field.

**Methods:** Publications related to ultrasound-induced BBB opening published from 1998 to 2020 were retrieved from Web of Science Core Collection. The search strategies were as follows: topic: (“blood brain barrier” OR “BBB”) AND topic: (ultrasound OR ultrason* OR acoustic* OR sonopora*). The document type was set to articles or reviews with language restriction to English. Three different analysis tools including one online platform, VOS viewer1.6.16, and CiteSpace V5.7.R2 software were used to conduct this scientometric study.

**Results:** A total of 1,201 valid records were included in the final analysis. The majority of scientific publication was produced by authors from North America, Eastern Asia, and Western Europe. *Ultrasound in Medicine and Biology* was the most prominent journal. The USA, China, and Canada were the most productive countries. Hynynen K, and Mcdannold N were key researchers with considerable academic influence. According to analysis of keywords, four main research directions were identified: cluster 1 (microbubbles study), cluster 2 (management of intracranial tumors), cluster 3 (ultrasound parameters and mechanisms study), and cluster 4 (treatment of neurodegenerative diseases). The current research hotspot has shifted from the basic research of ultrasound and microbubbles to management of intracranial tumors and neurodegenerative diseases. Burst detection analysis showed that Parkinson's disease, doxorubicin, gold nanoparticle, glioblastoma, gene therapy, and Alzheimer's disease may continue to be the research frontiers.

**Conclusion:** Ultrasound-induced BBB opening research is in a period of robust development. This study is a starting point, providing a comprehensive overview, development landscape, and future opportunities of this technology, which standout as a useful reference for researchers and decision makers interested in this area.

## Introduction

Advances in the knowledge of basic neuroscience, neuropharmacology, and nanomaterials have resulted in the availability of several novel therapeutic agents/genes that may be helpful to treat many central nervous system (CNS) diseases such as neurodegenerative, traumatic, inflammatory, and neoplastic diseases (Wong et al., 2012; Tian et al., 2019; Lochhead et al., 2020). The blood-brain barrier (BBB), located at the brain capillary endothelium, is a physiological barrier that separates the brain extracellular fluid from peripheral bloodstream and protects the brain from various circulating toxins and potentially harmful compounds (Cardoso et al., 2010). Nevertheless, it is also a major obstacle that restricts the access of therapeutic compounds to the CNS, imposing biochemical and size restrictions on the passage of molecules (Wong et al., 2019; Pandit et al., 2020).

A safe and effective technique of opening or circumventing the barrier temporarily could aid in the delivery of even large molecules, such as therapeutic agents, antibodies, neurotrophic factor, and cytokines, directly to brain pathology. Various strategies have been investigated, including direct intracranial infusion (Banks, 2016), hyperosmotic solutions (Kroll and Neuwelt, 1998), convection-enhanced delivery (Zhan and Wang, 2018), nose-to-brain pathways (Lochhead and Thorne, 2012; Su et al., 2020), and so on. However, these approaches have always been limited by the lack of selectivity of the target site, safety concerns, and a failure to achieve sufficient brain tissue concentrations of delivered compounds, and thus have not gained widespread acceptance in clinical practice (Banks, 2016; Zhang et al., 2016). Take hyperosmotic solutions as an example, although it is one of clinically validated methods that is able to enhance uptake of antineoplastic agents into the brain tumors, available research evidence indicates that the use of such reagents may cause structural alterations of endothelial cells, disruption of intercellular junctional complexes, macrophage accumulation, and glial activation (Wilhelm et al., 2008; Zhang et al., 2016).

In addition to the methods mentioned above, a physical BBB opening technique with ultrasound has aroused broad attention from researchers worldwide and has been extensively discussed in the literature in recent decades (Liu et al., 2010a,b). Nowadays, substantial evidence has demonstrated that focused ultrasound (FUS) coupled with microbubbles (1–10 μm in diameter) could lead to transient, focal, and reproducible BBB opening, thus enabling the therapeutic agents/genes across the BBB without long-term deficits in barrier function (Hernot and Klibanov, 2008; Sheikov et al., 2008; Dauba et al., 2020). The safety of ultrasound-induced BBB opening has been validated with magnetic resonance imaging (MRI) and histology results in a number of rodent models (Hynynen et al., 2001; Kinoshita et al., 2006; Leinenga et al., 2016). To date, this technique has shown efficacy in numerous preclinical studies with a wide range of agents that normally cannot be able to go through the BBB. In a series of clinical trials of this technique for the treatment of patients involved with brain tumors, Alzheimer's disease or epilepsy has been completed or ongoing worldwide (Alkins et al., 2016; Chen et al., 2019; Beccaria et al., 2020; D'Haese et al., 2020; Rezai et al., 2020). Concomitantly, numerous studies have investigated the potential mechanisms of ultrasound-induced BBB opening, and the mechanical ultrasound effects including inertial and stable cavitation, possibly play the most critical role in this process (Sheikov et al., 2004, 2008). More specifically, when microbubbles are subjected to ultrasound excitation, the bubbles expand/contract or even collapse (a phenomenon called cavitation), generating mechanical stresses on the capillary walls, transient disintegration of tight junctions, and temporary BBB opening (Lin et al., 2020). Often when optimal ultrasound exposure parameters are given, opening of BBB can be performed repeatedly without brain tissue damage.

Given the importance of this technology, a considerable number of scholars and academic journals have focused on reviewing relevant literature to summarize the current status of ultrasound-induced BBB opening research in recent years. Nevertheless, it is remarkable that much of this effort has only centered on specific subfields of ultrasound-induced BBB opening, with conclusions being drawn from systematic reviews or descriptive analysis (Hernot and Klibanov, 2008). For instance, most reviews have paid attention to a certain aspect of ultrasound-induced BBB opening for drug delivery (Leinenga et al., 2016), gene delivery (Timbie et al., 2015), immune modulation, or immune therapeutic delivery (Beccaria et al., 2021). There have been few reports that have focused on the scientometric perspective of ultrasound-induced BBB opening research.

Different from systematic reviews, scientometric analysis is a quantitative method that combines information visualization technology and mathematical and statistical methods to evaluate the contributions of authors, institutions, countries, and journals in specific fields (Rizzi et al., 2021; Yeung et al., 2021). It primarily focuses on the metrological properties of literature, like publication numbers, citation frequency, and cooperative relationships, so that the researchers can identify core entities and development trends in a specific subject or research domain and provide new insights and directions for future research. Currently, a variety of visualization tools including HistCite (Guo et al., 2020), VOS viewer (van-Eck and Waltman, 2010), R-bibliometrix (Ke et al., 2020), and CiteSpace (Synnestvedt et al., 2005) are freely available for scientometric analysis, and it has been widely employed in various areas, such as artificial intelligence (Guo et al., 2020), nanomedicine (Teles et al., 2018), stem cells (Zhao et al., 2018), immune molecules (Wang et al., 2020), and ethnopharmacology (Yeung et al., 2018). However, to the best of our knowledge, no existing study has yet specifically analyzed the knowledge structure and research frontiers in the field of ultrasound-induced BBB opening. Consequently, the current study is the first attempt to fill this research gap.

Based on the aforementioned background, this study conducts a scientometric analysis of ultrasound-induced BBB opening on the basis of published literature from 1998 to 2020. This study aimed to (i) summarize the current research trends of this field, (ii) determine the knowledge structure including the major academic groups and cooperation networks, (iii) identify the main research directions, especially the main clusters, and (iv) analyze the hotspots and frontiers of this technique to guide future research.

## Materials and Methods

### Data Source

Data from Science Citation Index Expanded (SCI-Expanded, 1998–present) of Web of Science Core Collection database (WoSCC) were used in this study. This database was chosen for three reasons. First, in contrast to other popular databases such as Google Scholar, PubMed, Scopus, or Embase, WOSCC is the most comprehensive and authoritative one across scientific disciplines (Chen et al., 2020, 2021). Second, it is a typical citation database containing literature abstracts and other relevant data such as citation and research collaboration information, facilitating for scientometric analysis (Yeung et al., 2021). Last but most importantly, it could directly provide reference files that met the specific format requirements as dictated by scientometric software such as VOS viewer and CiteSpace. Otherwise, an additional process is required to file format conversion if downloaded from other databases. As a consequence, WOSCC is recognized as one of the most suitable online databases for scientometric analysis (Zhao et al., 2018; Ke et al., 2020; Wu et al., 2021b). In addition, it could provide sufficient data that serve the purpose of this study.

### Data Collection

We performed online retrieval on February 20, 2021, from http://lib.tmu.edu.cn/ The following retrieval strategy was developed: topic: (“blood brain barrier” OR “BBB”) AND topic: (ultrasound OR ultrason* OR acoustic* OR sonopora*). Timespan: 1998 to 2020. Also, the document type was set to articles or reviews with language restriction to English. By means of the search field “topic,” WoSCC is able to recognize publications presenting the indicated phrases or their derivatives in the title, abstract, author keywords, or keywords plus. Wildcard “*” indicates any group of characters or no character in a pattern (for instance, “ultrason*” would also return “ultrasonography” or “ultrasonication”).

### Data Extraction

**Figure 1:**
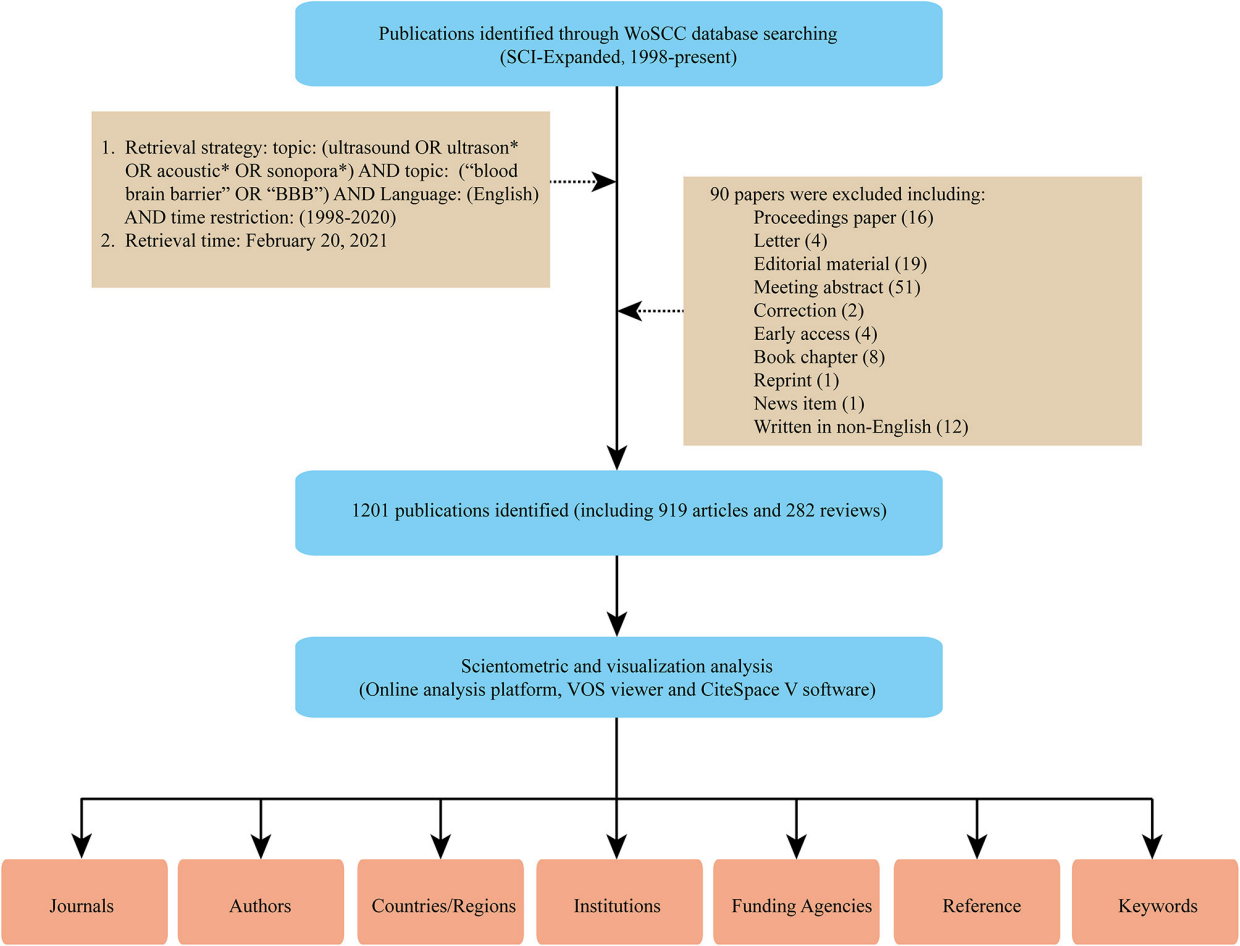
Flow diagram of literature search, screening, and analysis.

Using the above retrieval strategies, 1,201 pieces of literature were identified. “Full Record and Cited References” of these records including titles, authors, abstracts, and cited references were exported in the form of plain text. Two researchers (WH and ZY) independently searched the literature and extracted and cross-checked the relevant data. The discrepancies were resolved by discussion with the corresponding author (YH and SZ). The specific literature screening process is shown in Figure 1.

### Data Analysis

#### Descriptive Statistic

The filtered TXT file was imported manually into Microsoft Excel 2019 for quantitative analysis. We mainly focused upon the indicators as follows: the annual number of publication and citation; outputs and citation frequency of journals, countries, institutions, and authors; Hirsch index (H-index) of countries and authors; and impact factor (IF) and quartile in category of journals. The “citation report” option of WoSCC was available to assess H-index and citation frequency. The H-index indicates that a scholar/country has published H papers, each of which has been cited at least H times (Hirsch, 2005). This index is widely used to evaluate the productivity and scientific impact of a researcher or a country, and it rises with the increasing academic rank. Journal's IF and ranking were obtained from the 2019 Journal Citation Reports (Clarivate Analytics http://clarivate.com/products/web-of-science). Apart from the outputs of countries, another indicator (the number of papers per million people calculated by following formula: total production number/population) was also introduced for further quantification analyses (Wu et al., 2021a). Latest demographic data (2019) were obtained from the World Bank official website (https://data.worldbank.org.cn/country/) and the website of the Central People's Government of the People's Republic of China (http://www.gov.cn/guoqing/2020-07/28/content_5530577.htm). Moreover, GraphPad Prism 8 and R language (3.6.2) were used for statistical analysis and data visualization.

#### Scientometric Analysis With VOS Viewer Software

VOS viewer software is a scientometric visualization tool developed by Professor Eck and Waltman from Leiden University in the Netherlands using the Java language, which could visualize the knowledge structure, regularity, and distribution of scientific publications (van-Eck and Waltman, 2010). It has been widely adopted for bibliometric analysis in a variety of scientific fields since its introduction (Wang et al., 2020; Yeung et al., 2021). In our study, the version of VOS viewer 1.6.16 (available at: http://vosviewer.com) was used to perform country/institution citation analysis, journal/author/reference co-citation analysis, and keyword co-occurrence analysis, and related knowledge maps were constructed.

VOS viewer is capable of providing three visualization maps: the network visualization map, the overlay visualization map, and the density visualization map (van-Eck and Waltman, 2010). In the network visualization map, each node corresponds to a parameter such as country, institution, journal, author, or keywords, and the diameter of its size is roughly proportional to the number of publications, citations, or occurrences. Terms are located more closely to each other if they often co-occur in the same publications and will be assigned automatically to the one cluster with the same colors, otherwise located further away from each other with different colors. The links between nodes represent the network connections, and the strength of the links can be assessed quantitatively with the indicator of total link strength (TLS), which is the sum of link strengths of the terms over all the other terms (Zhao et al., 2018; Chen et al., 2021).

As for the overlay visualization map, the size, location, and links of the nodes have the same meaning as explained above, but unlike those, it could perform a timeline analysis of the terms. More specifically, the color of a term is determined by its average appearing year (AAY). The nodes coded with purple and blue color represented the terms that appeared relatively earlier upon time course before or around 2013, whereas keywords that appeared around 2015 were coded with green color, and those frequently used around or after 2017 appeared in yellow. While in the density visualization map, the darker color represents the higher frequency of the appearance.

#### Scientometric Analysis With CiteSpace Software

CiteSpace V (Version 5.7 R2, downloaded from http://cluster.cis.drexel.edu/) is another information visualization software tool created by Professor Chaomei Chen (Synnestvedt et al., 2005). In the present study, this software was implemented for constructing network visualization of author/institution cooperation analysis, co-occurring network of subject categories, as well as detecting the references and keywords with the strongest citation bursts (Zhong et al., 2020; Yan et al., 2021). Based on varying settings of time slices and thresholds, more pertinent conclusions can be drawn. The initial parameter settings of CiteSpace were as follows: time slicing (1998–2020), years per slice (1 year), node type (author, subject categories, institution, or keywords), links (strength: cosine, scope: within slices), and selection criteria (top 50 or 100).

Network maps generated by CiteSpace were also composed of links and nodes. The nodes normally represented the terms of author, subject categories, or institution, whereas links represent co-authorship between these nodes. The betweenness centrality (BC) is an important indicator that can unveil the importance of a node in the network, and the higher BC the node has, the larger impact the node has in the map (Chen, 2006). As for the burst detection of references and keywords, it actually relied on Kleinberg's algorithm, which can recognize the sharp increases of scientific activities over limited temporal duration and capture the increasing research interest in a specific research field (Kleinberg, 2003).

#### Scientometric Analysis With an Online Platform

Apart from the above methods, an online scientometric analysis platform (https://bibliometric.com/) was also used to perform international collaboration between countries.

### Research Ethics

No ethical approval was required, since all data used in this manuscript were obtained from public databases. No human participants and/or animals were involved in this study.

## Results

### Analysis of Publications and Citations

**Figure 2:**
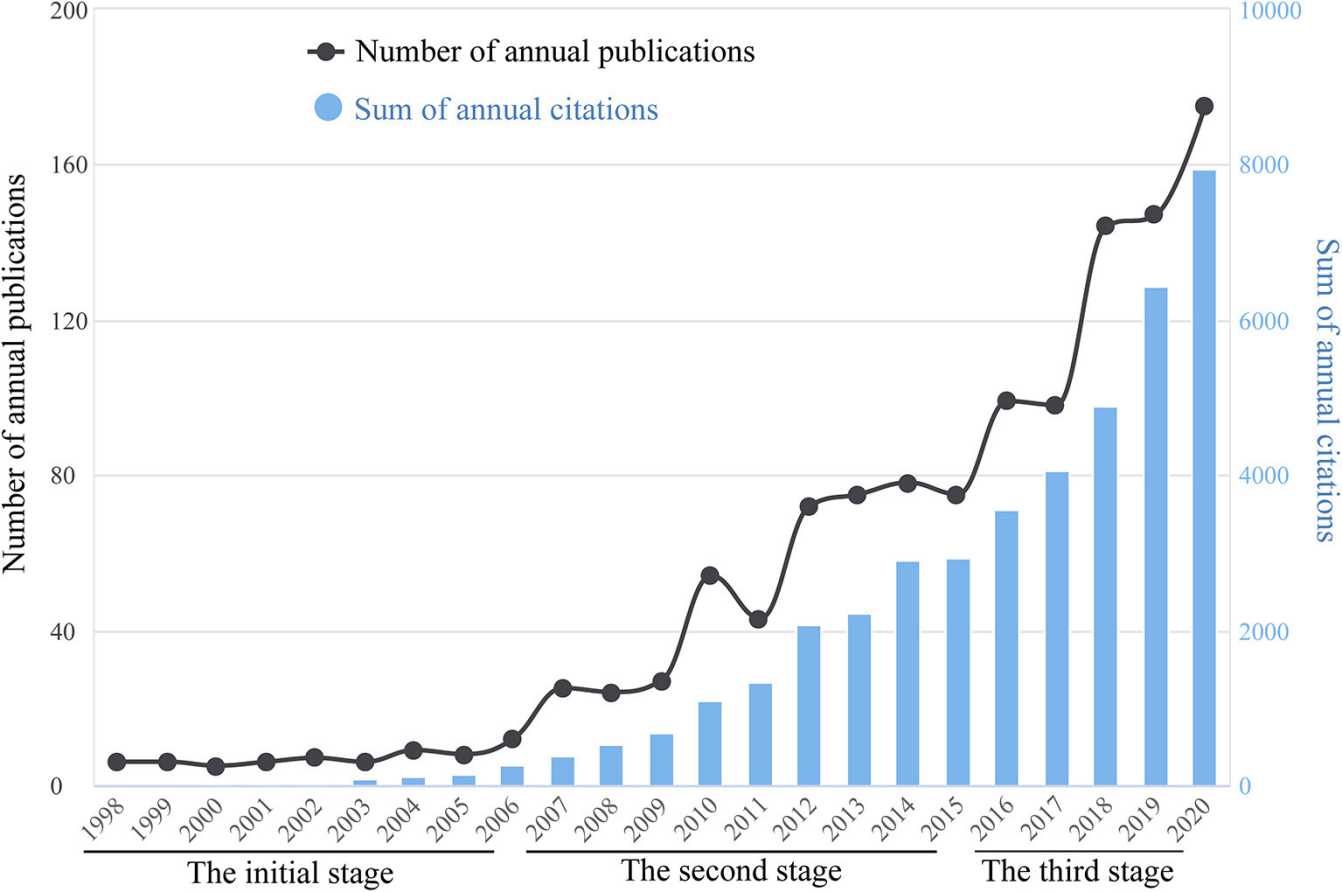
The number of published articles per year and summed total citations of annual publications related to ultrasound-induced BBB opening research from 1998 to 2020.

Of 1,291 records returned from the initial literature search, only 1,201 publications including 919 original research articles and 282 reviews were included in the final analysis (Figure 1). The cumulative total citations for all articles (average citations per item: 35.54) and that after the removal of self-citations were 42,684 and 28,500, respectively. The number of published articles per year and summed total citations of annual publications from 1998 to 2020 is shown in Figure 2. Research on ultrasound-induced BBB opening was roughly divided into three stages: the initial stage from 1998 to 2006, and the second stage from 2007 to 2015, and the third stage from 2016 to 2020.

### Analysis of Journals

**Figure 3:**
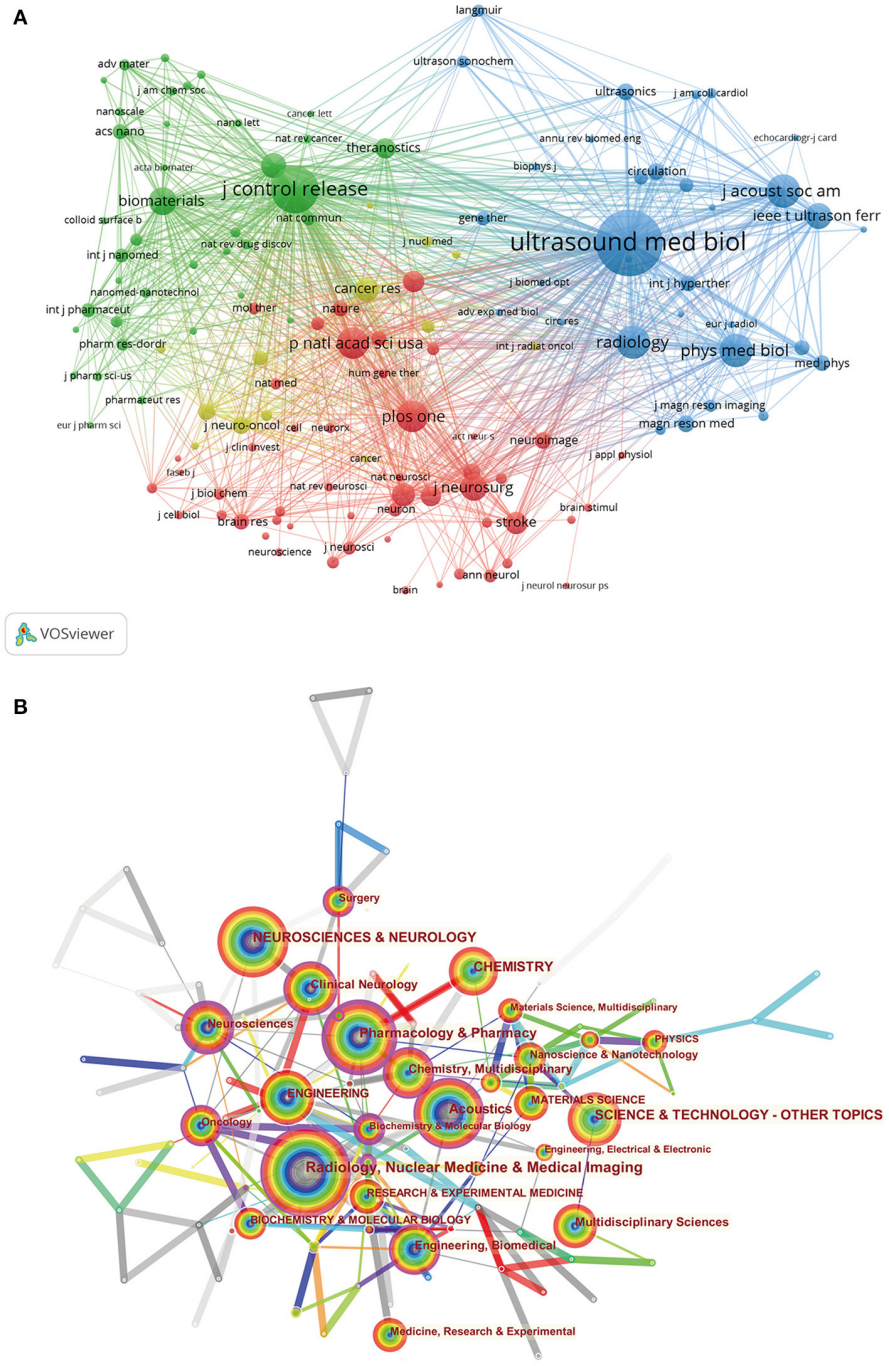
**(A)** The network visualization map of journal co-citation analysis by VOS viewer. **(B)** The co-occurrence network of subject categories was created with CiteSpace software.

We have listed the top 10 most productive journals in Table 1. *Ultrasound in Medicine and Biology* published the greatest number of articles/reviews, followed by *Journal of Controlled Release* and *Physics in Medicine and Biology*. According to the 2019 JCR reported, the IF of all the top 10 journals ranged from 2.514 (*Ultrasound in Medicine and Biology*) to 13.3 (*Advanced Drug Delivery Reviews*). Figure 3A illustrated the network visualization map of journal co-citation analysis. Only journals with a minimum number of 100 citations were depicted. There were 133 nodes and 8,765 links in the network map, and the top 3 with the largest TLS were *Ultrasound in Medicine and Biology, Journal of Controlled Release*, and *Journal of the Acoustical Society of America*.

**Table 1:** The top 10 productive journals that published articles on ultrasound-induced BBB opening.

**Ranking**	**Journal title**	**Country**	**Output [*n* (%)]**	**IF (2019)**	**Quartile in category (2019)**
1	Ultrasound in Medicine and Biology	USA	84 (6.99%)	2.514	Q1
2	Journal of Controlled Release	Netherlands	65 (5.41%)	7.727	Q1
3	Physics in Medicine and Biology	England	44 (3.66%)	2.883	Q2
4	Scientific Reports	England	44 (3.66%)	3.998	Q1
5	Plos One	USA	33 (2.75%)	2.74	Q2
6	Theranostics	Australia	31 (2.58%)	8.579	Q1
7	IEEE Transactions on Ultrasonics Ferroelectrics and Frequency Control	USA	28 (2.33%)	2.812	Q1
8	Expert Opinion on Drug Delivery	England	16 (1.33%)	4.838	Q1
9	Advanced Drug Delivery Reviews	Netherlands	15 (1.25%)	13.3	Q1
10	Journal of Neurosurgery	USA	14 (1.17%)	3.968	Q1

### Analysis of Subject Categories

According to the classification of subject categories in WoSCC, the top 15 research areas covered by the leading journals were illustrated in Supplementary Figure 1. Results obtained indicated that Radiology/Nuclear medicine/Medical imaging, Pharmacology/Pharmacy, and Neurosciences/Neurology were the top three popular research categories in this field. The subject categories assigned to the publications in our dataset were also analyzed by using CiteSpace, and one comprehensive network consisting of nodes representing co-occurrence subject categories was mapped in Figure 3B.

### Countries/Regions Analysis

**Figure 4:**
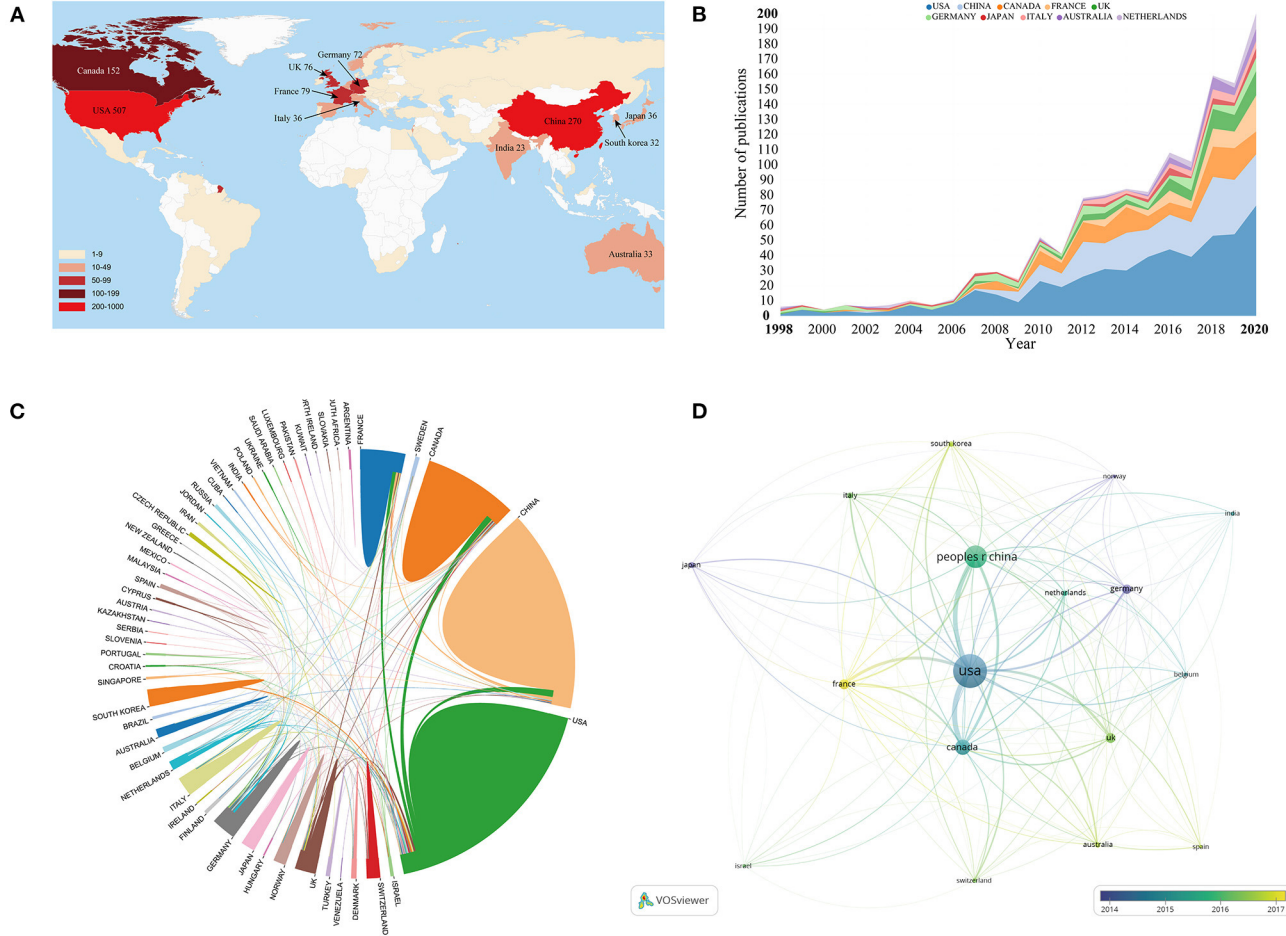
**(A)** Geographic distribution maps based on the total publications of different countries. **(B)** The annual number of publications from the top 10 countries between 1998 and 2020. **(C)** An analysis of international cooperation among countries. **(D)** Citation network map of countries generated by using VOS viewer software.

Fifty-six countries contributed to the publications on ultrasound induced BBB opening research. As displayed in Table 2, the top three countries with the most articles/reviews were the USA, China, Canada, and the other countries published <100 documents. After adjusting by population size, Canada was on top with 4.04 papers per million people, followed by Switzerland (3.03) and Norway (2.43). In the aspect of H-index, the USA, Canada, and China were also ranked as the top 3 countries. A geographical distribution map based on the total publications of different countries was presented in Figure 4A. The majority of scientific publication in this field was produced by authors from North America, Eastern Asia, and Western Europe. Our comparison of the total amount of publications from the three regions revealed that North American authors published ~2 times higher than Western European authors and 1.6 times higher than Eastern Asian authors. This result demonstrated that researchers from North American countries were more active than those in Western European and Eastern Asian countries. Figure 4B illustrated the annual number of publications from the top 10 countries between 1998 and 2020.

**Table 2:** The top 20 productive countries in the publications concerning ultrasound-induced BBB opening research.

**Ranking**	**Countries**	**Output [*n* (%)]**	**Population (in millions)**	**Number of papers per million people**	**Optimized ranking**	**H-index**	**TLS**	**Citations**
1	USA	507 (42.22%)	328.24	1.54	5	78	9,947	23,474
2	China	270 (22.48%)	1,429.47	0.19	19	47	4,384	7,108
3	Canada	152 (12.66%)	37.59	4.04	1	50	5,535	6,876
4	France	79 (6.58%)	67.06	1.18	10	23	2,149	1,729
5	UK	76 (6.33%)	66.83	1.14	11	23	1,370	2,360
6	Germany	72 (6%)	83.13	0.87	14	27	1,120	2,087
7	Italy	36 (3%)	60.3	0.60	16	13	618	644
8	Japan	36 (3%)	126.26	0.29	18	13	337	812
9	Australia	33 (2.75%)	25.36	1.30	9	14	680	919
10	South Korea	32 (2.66%)	51.71	0.62	15	12	597	477
11	Netherlands	31 (2.58%)	17.33	1.79	4	16	790	965
12	Switzerland	26 (2.16%)	8.57	3.03	2	12	549	426
13	India	23 (1.92%)	1366.42	0.02	20	11	191	878
14	Spain	15 (1.25%)	47.08	0.32	17	7	267	349
15	Belgium	13 (1.08%)	11.48	1.13	12	9	409	998
16	Norway	13 (1.08%)	5.35	2.43	3	10	321	271
17	Israel	12 (1%)	9.05	1.33	8	8	158	439
18	Sweden	9 (0.75%)	10.29	0.87	13	7	87	217
19	Denmark	8 (0.67%)	5.82	1.37	7	7	19	391
20	Finland	8 (0.67%)	5.52	1.45	6	6	95	241

*TLS, total link strength. Ranking: according to the number of total papers. Optimized ranking: according to the number of papers per million people*.

*Publications from Taiwan, Hong Kong, and Macau were assigned to China, and those from England, Northern Ireland, Scotland, and Wales were reclassified to the UK*.

An analysis of international cooperation among countries was also conducted in Figure 4C. Active collaboration was observed between prolific countries. For example, the USA collaborated closely with Canada, China, and France. China, France, and the UK demonstrated active cooperation as well. A citation network map was created for countries with at least 10 documents (Figure 4D). Of the 17 countries that met this threshold, the top three with the largest TLS were the USA, Canada, and China.

### Funding Agencies Analysis

**Figure 5:**
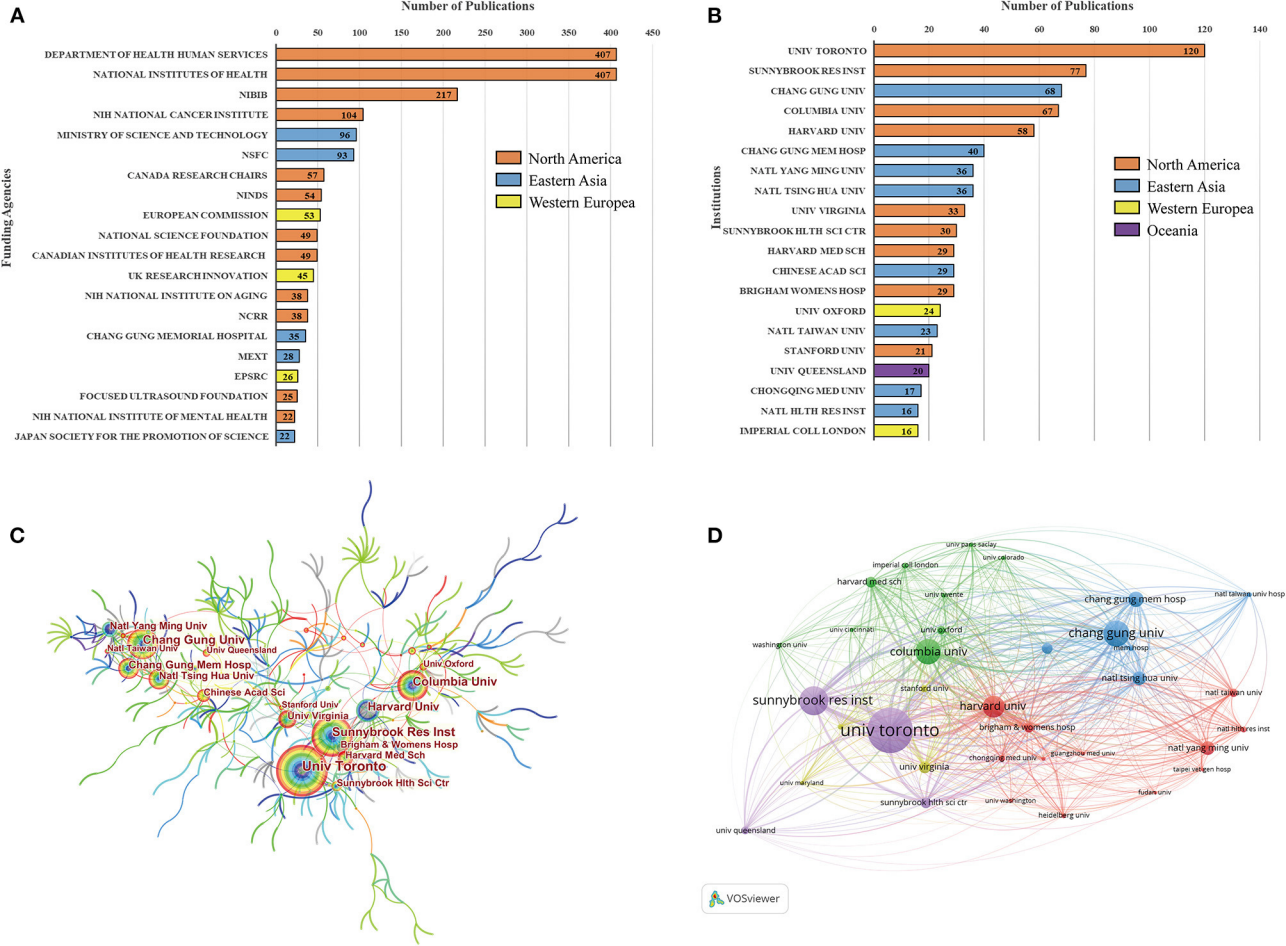
**(A)** The top 20 funding agencies for the output of ultrasound-induced BBB opening research. NIBIB, NIH National Institute of Biomedical Imaging Bioengineering; NSFC, National Natural Science Foundation of China; NINDS, NIH National Institute of Neurological Disorders Stroke; NCRR, NIH National Center for Research Resources; MEXT, Ministry of Education Culture Sports Science and Technology Japan; EPSRC, Engineering Physical Sciences Research Council. **(B)** The top 20 institutions with the most publications of ultrasound-induced BBB opening research. **(C)** The institutional cooperation map created with CiteSpace software. **(D)** Institution citation analysis was performed with VOS viewer.

Figure 5A lists the top 20 funding agencies for the output of ultrasound-induced BBB opening research. Among them, the fund project of The United States Department of Health and Human Services (HHS) tied in first place with the National Institutes of Health (NIH), all of which support 407 studies, respectively. Also, in terms of regional distribution, funding agencies from North America sponsored the highest number of studies.

### Institutions Analysis

As can be seen from Figure 5B, the top 20 institutions accounted for 789 (65.7%) of all literature in this field. Among which nine institutions originated from North America, eight from Eastern Asia, and the remaining three came from Western Europe and Oceania. The most prolific individual institution in terms of number of publications was Univ Toronto, followed by Sunnybrook Res Inst and Chang Gung Univ. From the institutional cooperation map in Figure 5C, Univ Toronto (BC = 0.17) was situated in a central position, and the density of the overall network is relatively low. Institution citation analysis was performed with VOS viewer. Publications originating from 35 institutions with the minimum number of 10 documents were selected and analyzed by VOS viewer (Figure 5D). The top three institutions with the largest TLS were as follows: Univ Toronto, Harvard Univ, and Sunnybrook Res Inst.

### Authors Analysis

**Figure 6:**
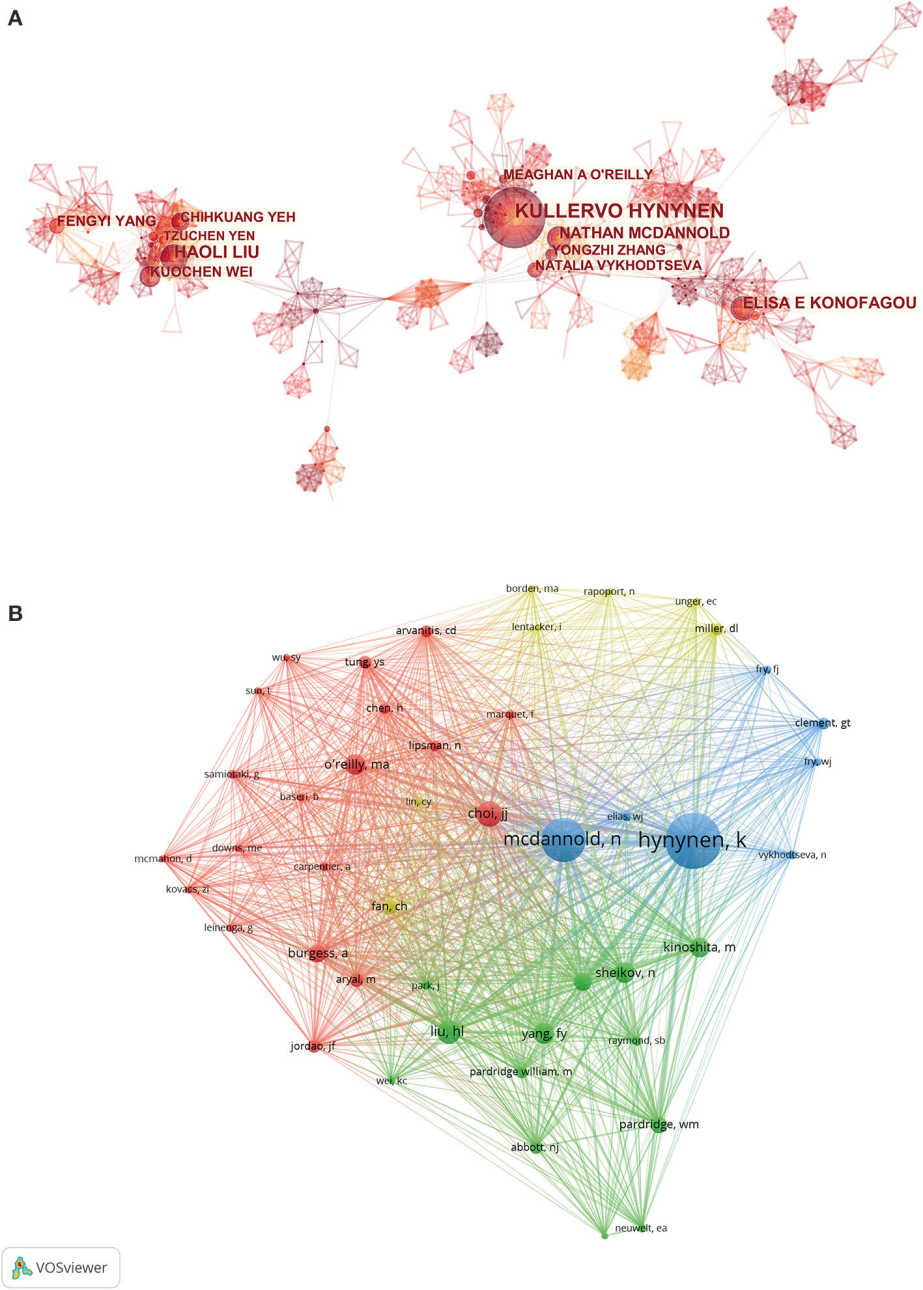
**(A)** The cooperation network visualization map among productive authors analyzed by CiteSpace. **(B)** The co-citation analysis of the authors carried out by using VOS viewer.

Table 3 lists the top 10 most prolific authors and co-cited authors in this field. Most of these authors came from the USA and China. Hynynen K from Brigham and Women's Hospital/Harvard Medical School, contributed the highest number of 126 papers, followed by Liu H L from Chang Gung Memorial Hospital/Chang Gung University, and Konofagou E E from Columbia University, with 62 and 51 publications, respectively. The cooperation relationship among productive authors was analyzed by CiteSpace. As shown in Figure 6A, the centrality index for each author was lower than 0.1, and quite small number of connection links were observed in this network map. The co-citation analysis of the authors was carried out using VOS viewer. As shown in Figure 6B, 46 authors with at least 100 citations were included in the analysis. The top three authors with the greatest TLS were Hynynen K, Mcdannold N, and Choi J J.

**Table 3:** The top 10 productive and most co-cited authors in ultrasound-induced BBB opening research.

**Ranking**	**Author**	**Output [*n* (%)]**	**H-index**	**Co-cited author**	**Country/region**	**TLS**	**Citations**
1	Hynynen K	126 (10.49%)	54	Hynynen K	USA	10,480	9,760
2	Liu H L	62 (5.16%)	30	Mcdannold N	USA	4,355	3,763
3	Konofagou E E	51 (4.25%)	31	Liu H L	China	4,233	2,701
4	Mcdannold N	50 (4.16%)	33	Konofagou E E	USA	3,315	2,496
5	Vykhodtseva N	34 (2.83%)	27	Vykhodtseva N	USA	3,258	2,659
6	Yang F Y	31 (2.58%)	14	Wei K C	China	2,371	1,668
7	Yeh C K	31 (2.58%)	20	Yeh C K	China	2,076	1,297
8	O'Reilly M A	29 (2.41%)	29	O'Reilly M A	Canada	1,996	1,121
9	Wei K C	29 (2.41%)	22	Yen T C	China	1,759	1,561
10	Zhang Y Z	26 (2.16%)	17	Choi J J	UK	1,654	1,284

*TLS, total link strength*.

### References and Co-cited References

**Figure 7:**
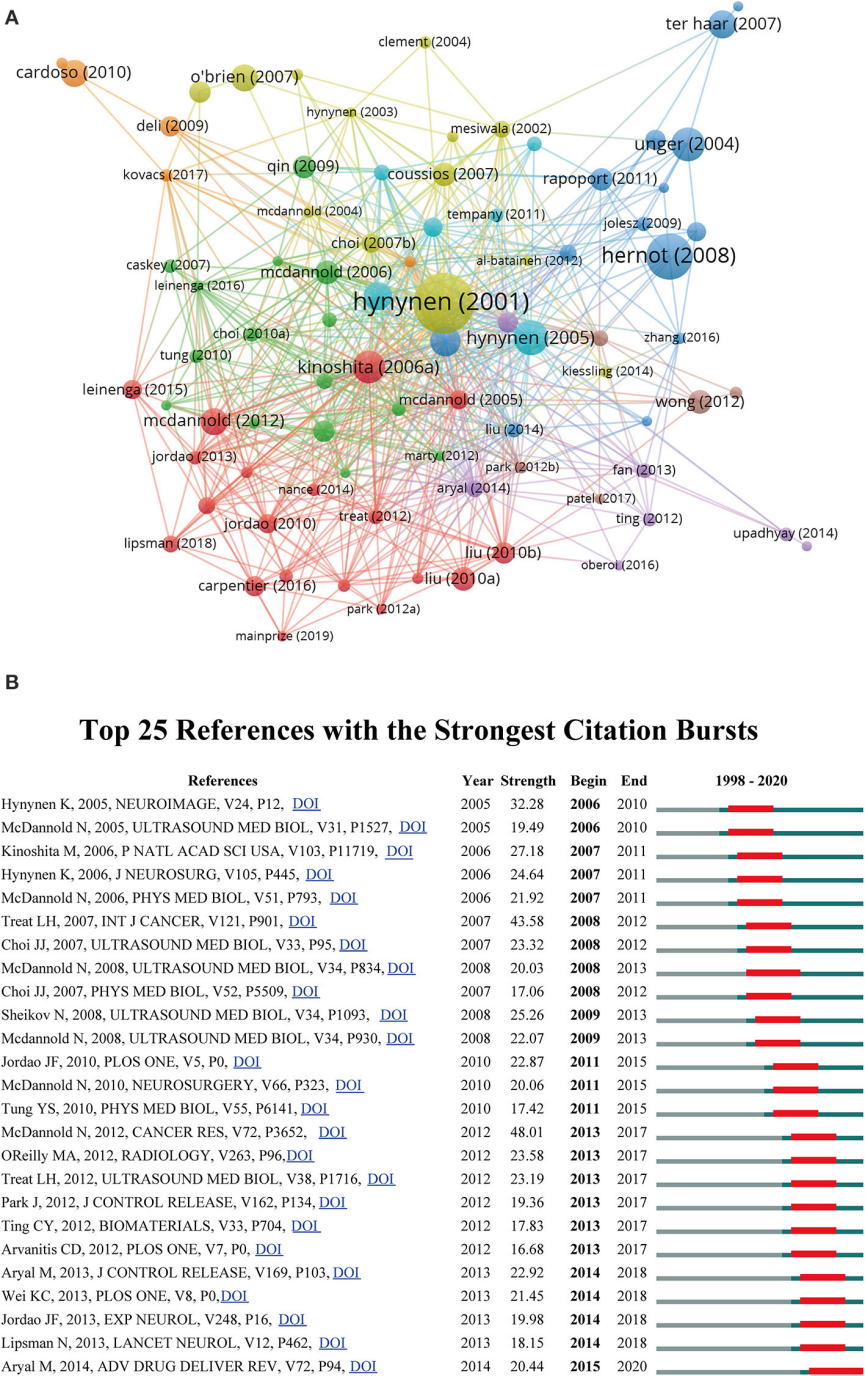
**(A)** Co-citation network visualization map of references generated by VOS viewer. **(B)** The top 25 references with the strongest citation bursts from 1998 to 2020.

The top 10 most co-cited original articles related to ultrasound-induced BBB opening research are given in Table 4. Only one of the top 10 included references was published after 2010, with the majority being published in or before 2010. All of them were co-cited more than 250 times. Moreover, references co-citation network visualization map generated by VOS viewer was also provided. As shown in Figure 7A, the reference of Hynynen et al. was located in central positions within the network. Figure 7B illustrates the top 25 references with the strongest citation bursts from 1998 to 2020. Reference with citation burst was first observed in 2006, which is due to an article by Hynynen et al. in 2005. The most recent burst appeared in 2015 and has lasted for 5 years until now.

**Table 4:** The top 10 original articles related to ultrasound induced BBB opening with the most citations.

**Ranking**	**Title**	**Total citations**	**Journal**	**First author**	**Year**	**Main conclusion**
1	Non-invasive MR imaging-guided focal opening of the blood-brain barrier in rabbits	842	*Radiology*	Hynynen K	2001	They confirmed that in the presence of ultrasound contrast agents, focused ultrasound was able to open the BBB consistently in a rabbit model, and this process could be monitored with magnetic resonance imaging.
2	Local and reversible blood-brain barrier disruption by non-invasive focused ultrasound at frequencies suitable for trans-skull sonications	441	*Neuroimage*	Hynynen K	2005	Their study has demonstrated that non-invasive BBB disruption was possible at a frequency of 0.69 MHz in a rabbit model.
3	Non-invasive localized delivery of herceptin to the mouse brain by MRI-guided focused ultrasound-induced blood-brain barrier disruption	416	*Proc Natl Acad Sci U S A*	Kinoshita M	2006	They reported that Herceptin could be delivered non-invasively into the mouse CNS by using an MRI-guided focused ultrasound BBB disruption technique.
4	Targeted delivery of doxorubicin to the rat brain at therapeutic levels using MRI-guided focused ultrasound	374	*Int J Cancer*	Treat L H	2007	They revealed that with the help of MRI-guided focused ultrasound, doxorubicin could be delivered non-invasively into CNS and achieved the therapeutic levels.
5	Cellular mechanisms of the blood-brain barrier opening induced by ultrasound in presence of microbubbles	339	*Ultrasound Med Biol*	Sheikov N	2004	They investigated the endothelial cell fine morphology after the BBB opening induced by ultrasound combined with microbubbles and proposed several possible mechanisms of transcapillary passage.
6	Temporary disruption of the blood-brain barrier by the use of ultrasound and microbubbles: safety and efficacy evaluation in rhesus macaques	320	*Cancer Res*	McDannold N	2012	They identified the safety, reliability, and controllability of ultrasound combined with microbubble technique in a clinically relevant animal model.
7	Targeted disruption of the blood-brain barrier with focused ultrasound: association with cavitation activity	282	*Phys Med Biol*	McDannold N	2006	They found that localized BBB disruption could be achieved with focused ultrasound combined with contrast agent without detecting inertial cavitation.
8	Magnetic resonance monitoring of focused ultrasound/magnetic nanoparticle targeting delivery of therapeutic agents to the brain	274	*Proc Natl Acad Sci U S A*	Liu HL	2010	A combined of focused ultrasound and magnetic targeting could deliver therapeutic magnetic nanoparticles across the BBB synergistically.
9	Blood-brain barrier disruption with focused ultrasound enhances delivery of chemotherapeutic drugs for glioblastoma treatment	252	*Radiology*	Liu HL	2010	They demonstrated that focused ultrasound in the presence of microbubbles could increase localized chemotherapeutic drug delivery to glioblastomas in rats.
10	Effect of focused ultrasound applied with an ultrasound contrast agent on the tight junctional integrity of the brain microvascular endothelium	250	*Ultrasound Med Biol*	Sheikov N	2008	This study provided the first direct evidence that ultrasound combined with contrast agent could cause disassembling of the BBB tight junction molecular structure.

### Keywords Co-occurrence Analysis

**Figure 8:**
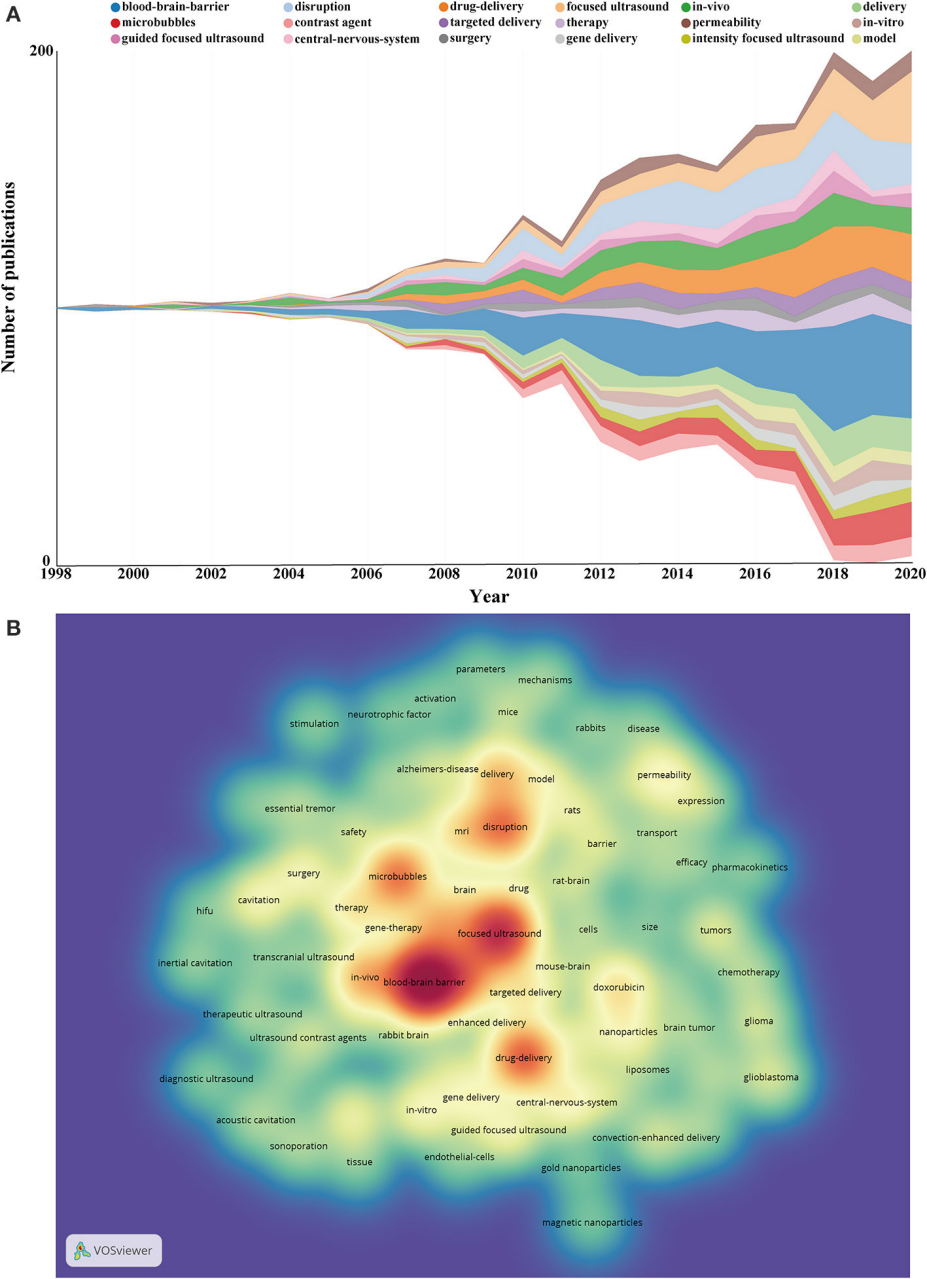
**(A)** The change pattern of annual keyword appearance frequency from 1998 to 2020. **(B)** A density visualization map was drawn with 82 included keywords by using VOS viewer.

**Figure 9:**
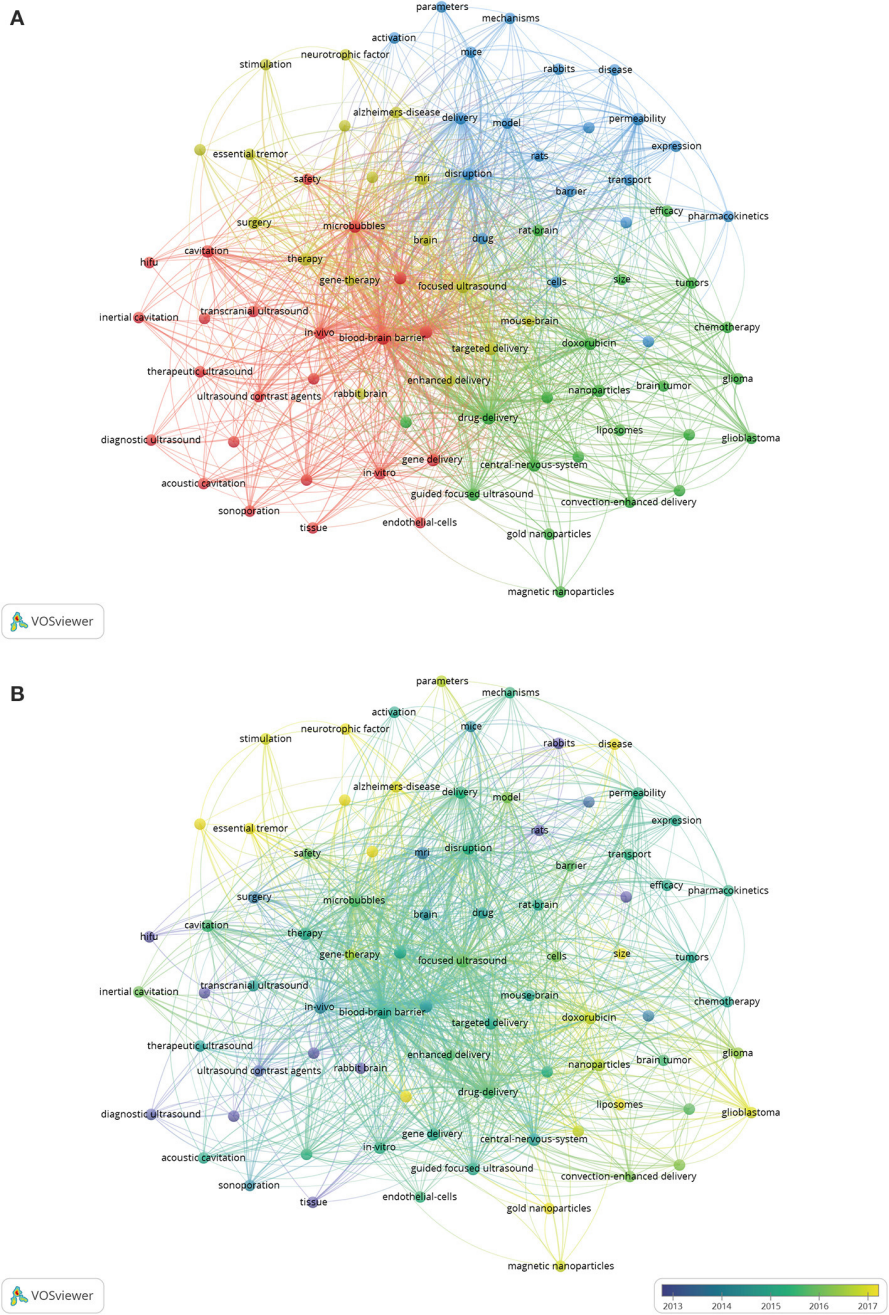
**(A)** The network visualization map of the 82 keywords generated by using VOS viewer software. All the keywords could be clustered into four categories: cluster 1 (microbubbles study, red nodes), cluster 2 (management of intracranial tumors, green nodes), cluster 3 (ultrasound parameters and mechanisms study, blue nodes), and cluster 4 (treatment of neurodegenerative diseases, yellow nodes). **(B)** The overlay visualization map of the 82 keywords generated by using VOS viewer software. The nodes coded with purple and blue color represented the keywords that appeared relatively earlier upon time course before or around 2013, whereas keywords that appeared around 2015 were coded with green color and those frequently used around or after 2017 appeared in yellow.

Research hotspots and frontiers of ultrasound-induced BBB opening can be identified by analyzing the results of keywords co-occurrence. The change pattern of annual keyword appearance frequency from 1998 to 2020 was illustrated in Figure 8A. The keyword co-occurrence visualization map was constructed with the VOS viewer software. A total of 4,740 keywords were extracted from the 1,201 papers, and 82 keywords which occurred at least 20 times were included. A density visualization map is shown in Figure 8B, “blood-brain barrier” was the keyword that appeared most frequently, followed by “focused ultrasound,” “drug-delivery,” and “microbubbles.” To further study the topic structure of ultrasound-induced BBB opening research, CiteSpace was also used to establish a visualization knowledge map of high-frequency keywords (Supplementary Figure 2). The network and overlay visualization map of the 82 keywords were also offered in Figure 9. As can be seen from Figure 9A, there were 82 nodes, 2,376 links, and a total link strength of 3,179. All the keywords could be clustered into four categories: cluster 1 (microbubbles study, red nodes), cluster 2 (management of intracranial tumors, green nodes), cluster 3 (ultrasound parameters and mechanisms study, blue nodes), and cluster 4 (treatment of neurodegenerative diseases, yellow nodes). Figure 9B was the overlay visualization map of the 82 keywords, which summarizes the keyword occurrences with a time zone perspective according to their AAY.

### Burst Keywords

**Figure 10:**
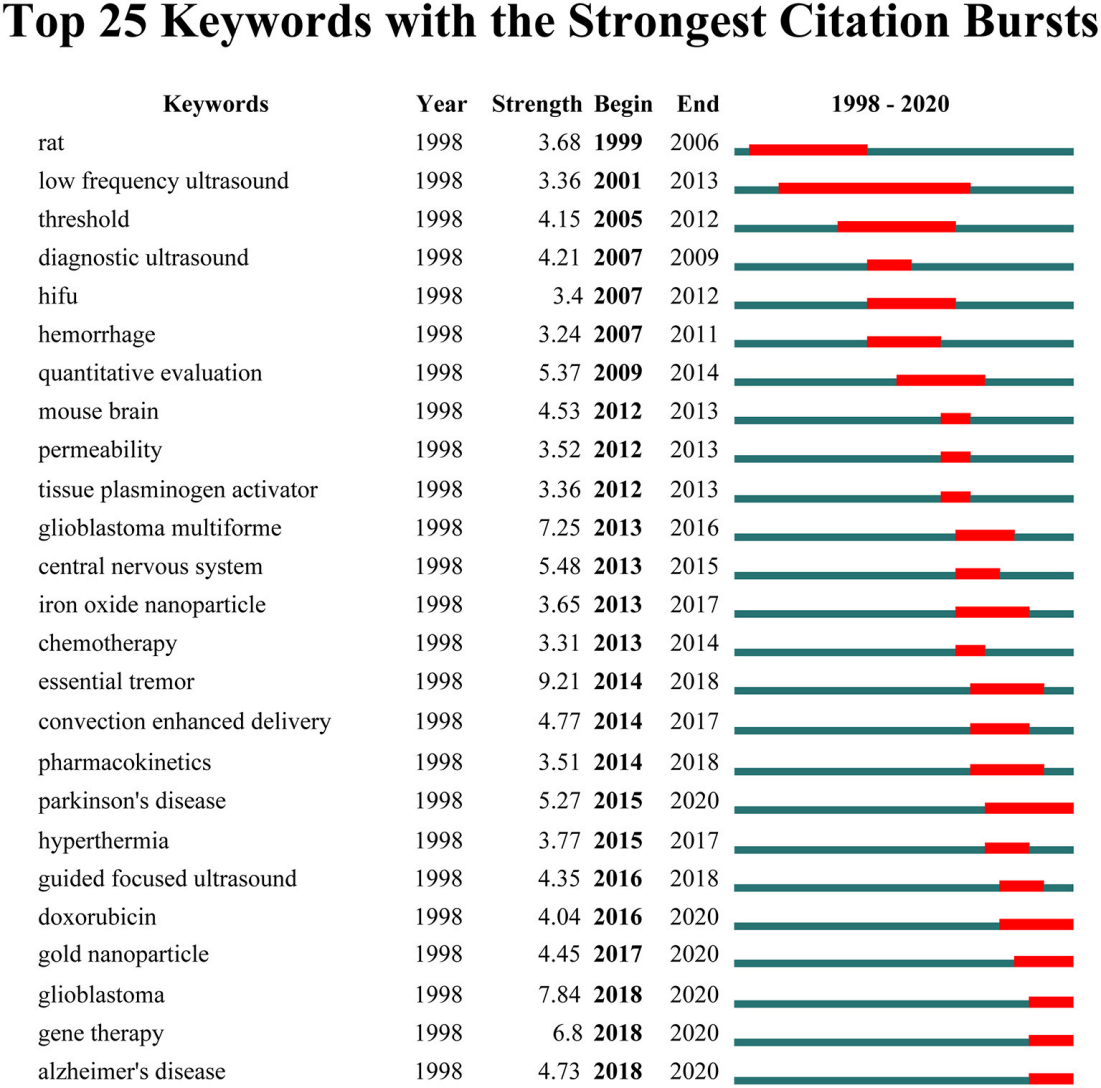
The top 25 keywords with the strongest citation bursts from 1998 to 2020.

Keywords with strong citation bursts were also captured through CiteSpace, and top 25 keywords with the strongest citation bursts are listed in Figure 10. During the initial stage from 1998 to 2005, studies of this domain have focused on animals experiments with rabbits and low frequency ultrasound. In the second phase from 2007 to 2015, diagnostic ultrasound, high-intensity-focused ultrasound (HIFU), quantitative evaluation, permeability, and chemotherapy became the research foci. The keywords that had strong bursts after 2015 were as follows: “Parkinson's disease (2015–2020),” “hyperthermia (2015–2017),” “guided focused ultrasound (2016–2018),” “doxorubicin (2016–2020),” “gold nanoparticle (2017–2020),” “glioblastoma (2018–2020),” “gene therapy (2018–2020),” and “Alzheimer's disease (2018–2020).” Notably, the burst of these five keywords including “Parkinson's disease,” “doxorubicin,” “gold nanoparticle,” “glioblastoma,” “gene therapy,” and “Alzheimer's disease” is still ongoing.

## Discussion

### Global Research Trends From 1998 to 2020

This paper is, to our knowledge, the first attempt to reveal the knowledge maps of global scientific publications related to ultrasound-induced BBB opening research from 1998 to 2020 by employing a scientometric analysis. Analyzing the data coming from the annual number of frequency of publications and citations, we found that research on ultrasound-induced BBB opening was roughly divided into three stages. The initial stage lasted for ~9 years, during which the annual number of related documents was <15. In the second stage, there was a slight annual increase in the number of publications, fluctuating right above 20 during this period. From 2016 onwards, the annual number of publications increased more steeply and exceeded 100 papers for the first time in 2018. In particular, the total number of publications in this stage accounted for over 55% of all included papers for the entire period. Interestingly, along with the changing trend of annual publication number, there has been also a similar growth pattern of summed citations. As we mentioned earlier, ultrasound-induced BBB opening is an exciting field with significant progress and scientific advances. Based on the current data, there has been a surge of interest in this BBB opening technique whether from the perspective of annual publication number or citation number. In addition, one may speculate that more studies on ultrasound-induced BBB opening will be published in the next few years.

### Knowledge Structure of Global Publications

#### Journals

Statistical analysis of journal distribution is helpful for scholars to quickly select the best-fit journals for publication of their research findings. It is not difficult to see that these journals identified in Table 1 are mainly regarding acoustics, medical imaging, pharmacology, biology, and medicine, and they may be the core journals that have been greatly welcomed by scientific researchers in this field. More related research results will be published in these journals in the future. In addition, among the top 10 journals, 90% were from Western European and North American countries, indicating that journals from Euro-American countries dominated in this field.

Co-citation analysis indicates the relationship between items based on the frequency they are cited together, with TLS being calculated to assess the total link strength (Zhao et al., 2018; Chen et al., 2021). The journal co-citation analysis showed that *Ultrasound in Medicine and Biology, Journal of Controlled Release*, and *Journal of the Acoustical Society of America* were the top three journals with the largest TLS. As we can see from the results, research findings related to ultrasound-induced BBB opening that are published in these journals, may have a greater chance to be cited and receive considerable attention. Of note, *Ultrasound in Medicine and Biology* ranked first with the largest TLS among co-cited journals; the position may be related to contributions of two landmark studies investigating the mechanism of ultrasound-induced BBB opening (Sheikov et al., 2004, 2008). In addition, Zheng et al. (2016) also have performed a bibliometric analysis to trace the global trends in micro/nano-bubble-related research from 1991 to 2014. They also found that *Ultrasound in Medicine and Biology* is the leading journal in this field with the highest H-index of 56. Whereas, Wu and colleagues have carried out a comprehensive bibliometric analysis of global research trends on ultrasound microbubble. *Ultrasound in Medicine and Biology* published the most papers in this field, accounting for 11.33% of the publications, and also had the highest BC, with a central value of 0.67. Therefore, we recommend that scholars in this domain should pay attention to the scientific outputs published by these journals to obtain recent advances.

#### Subject Categories

Results obtained from Figure 3B indicated that Radiology/Nuclear medicine/Medical imaging, Pharmacology/Pharmacy, and Neurosciences/Neurology were the top three popular research categories in this field. Meanwhile, it also suggests that research on ultrasound-induced BBB opening has covered multidisciplinary knowledge. Interdisciplinary collaboration among different fields may help to improve scientific work and maximize the potential.

#### Countries/Regions

In terms of the number of publications, the USA, China, and Canada have made the greatest contribution within this important field. The USA has always been a leader in many domains of neuroscience, which has been confirmed by other scientometric studies (Devezas, 2021; Wu et al., 2021b; Yan et al., 2021). However, after adjusting by population size, Canada was on top with 4.04 papers per million people, while China was only in the 19th position. Therefore, despite the great progress in terms of total publications, there is still a certain gap in China compared with developed countries. Notably, China ranked among the top three with regard to the H-index, which was not common in other scientometric analysis because the quality of most studies was not high (Ye J. et al., 2018; Yao et al., 2020). However, the results of the present study showed that the overall quality of the research papers in China had been greatly improved in this field.

Figure 4B illustrates the annual number of publications from the top 10 countries between 1998 and 2020, which reflects the change trend among different countries in this field. It can be seen that the USA has been at the forefront of this field for two decades, while China started a process of rapid development after 2010. Furthermore, a citation network map was created for countries with at least 10 documents. It is interesting to note that all the top three countries with the largest TLS were also the top three countries with the most publications. This outcome further proved the central position of them in this field. In addition, as also can be seen from this citation map, the nodes that represent Germany, Japan, and Norway are given purple color (with earlier average appearing years), indicating that these countries were more active in the early stage of ultrasound-induced BBB opening research. France is yellow with AAY of 2,017.13, implying that it is newly active in this field.

#### Funding Agencies

As we all know that the development of innovative technologies and approaches requires a large amount of financial resources, we obtained information about the funding agencies for these researches. The principal funding supporters were national foundations, for example: HHS, NIH, NIBIB, and NSFC. From the distribution of the funding agencies, North America and Eastern Asia sponsored the highest number of studies. Among them, the fund project of HHS tied in first place with NIH, all of which support 407 studies, respectively. Based on the above results, one may find that the USA occupied the absolute dominant position in this field which cannot be separated from the adequate financial input. Of course, adequate funding can also attract a wider variety of researchers and institutions devote more work to this arena, which is a mutually reinforcing process.

#### Institutions

As for analysis of institutions, in the list of top 20 productive institutions, nine institutions originated from North America, eight from Eastern Asia, and the remaining three came from Western Europe and Oceania. This could also serve as an important reason for a large number of papers published in these regions. On the other hand, this illustrated that the formation of top-notch research institutions is a key way to improve academic influence of a country. From the institutional cooperation map, it can be seen that Univ Toronto (BC = 0.17) was situated in a central position.

Institution citation analysis showed that the top three institutions with the largest TLS were Univ Toronto, Harvard Univ, and Sunnybrook Res Inst. Surprisingly, the total number of publications of Harvard Univ from 1998 to 2020 were the lowest (58) among the top five institutions, but it still had the higher academic influence, indicating that Harvard Univ had performed higher-quality studies in this area. Additionally, summarizing the abovementioned results, it can be concluded that the Univ Toronto in Canada was the most productive and influential institution and collaborated closely with many other institutions worldwide. Foreseeably, apart from acquiring more financial support, there is a strong possibility for some important findings related to ultrasound-induced BBB opening reported by these abovementioned institutions in the future. Even more significant, these findings enable researchers who are interested in ultrasound-induced BBB open to identify leading research institutions and scholars of this field and thus to seek out potential academic collaboration.

#### Authors

The earliest developments and research on ultrasound-induced BBB opening were initially conducted by several American scholars who focused on the mechanism and potential clinical applications of ultrasound-induced BBB opening (Hynynen et al., 2001, 2005; Kinoshita et al., 2006; McDannold et al., 2006). Therefore, the researchers from the USA were the main driving force, as evidenced by four of the top 5 most productive authors being from the USA. Hynynen K from Brigham and Women's Hospital, Liu H L from Chang Gung Memorial Hospital/Chang Gung University (Liu et al., 2010a,b), and Konofagou E E (Konofagou, 2012; Pouliopoulos et al., 2020) from Columbia University contributed the most publications. However, in terms of authors' cooperation analysis, the centrality index for each author was <0.1, and quite a small number of connection links were observed in this network map, which reflected that there was little collaboration between different research teams. Hence, more attention should be paid to the international as well as interdisciplinary collaboration between research groups to improve the development of this field.

In addition, the top 10 most co-cited original articles related to ultrasound-induced BBB opening research are given in Table 4. Highly cited articles generally refer to the high-quality studies with great impact in terms of innovation and discovery in a certain field. These publications are also regarded as the essential readings for scholars prepared to commit to the work. The most highly cited reference in our dataset is an article by Hynynen et al. in 2001. In this study, they confirmed that in the presence of ultrasound contrast agents, FUS was able to open the BBB consistently in a rabbit model, and this process could be monitored with magnetic resonance imaging (Hynynen et al., 2001). Notably, as the first author or co-author, Hynynen K has also participated in the eight studies among the top 10 most co-cited original articles (Kinoshita et al., 2006; McDannold et al., 2006, 2012; Treat et al., 2007). This indicates that he and his team have made significant scientific contributions and a profound impact on other researchers in the field. This was further confirmed by co-citation of authors and co-citation network visualization map of references. In the co-citation network map of reference, the reference by Hynynen et al. (2001) was located in central positions within the network. As for co-citation analysis of authors, Hynynen K was the author with the largest TLS.

### Research Clusters and Focus of Global Publications

A total of 4,740 keywords were extracted from the 1,201 papers. For 82 keywords which occurred at least 20 times, blood-brain barrier, focused ultrasound, drug delivery, and microbubbles were the keywords that appeared most frequently, which was concordant with the subject of the present study. In scientometric studies, co-occurrence analysis of keywords was often used to identify major research clusters and focus in a research field. The current results showed that all the keywords could be clustered into four categories: cluster 1 (microbubbles study) was the largest cluster mainly related to microbubbles, blood-brain barrier, ultrasound contrast agents, cavitation, and *in vitro*. Cluster 2 (management of intracranial tumors) contained 22 keywords, and the primary keywords were drug delivery, guided focused ultrasound, doxorubicin, chemotherapy, glioma, and glioblastoma. Cluster 3 includes 20 keywords, mainly focused on ultrasound parameters and mechanisms including the following keywords: disruption, delivery, permeability, transport, mechanisms, and parameters. As for cluster 4 (treatment of neurodegenerative diseases), the prominent keywords were focused ultrasound, Alzheimer's disease, Parkinson's disease, gene therapy, and neurotrophic factor.

#### Cluster 1: Study of Microbubbles

Microbubble is a small vesicular structure comprising a gas core surrounded by a biocompatible shell built up by phospholipids, albumin, or polymer. As we all know, microbubble preparations were originally developed and exploited as ultrasound contrast agents, which were used for the enhancement of ultrasound imaging and detection, due to their ability to generate an enhanced echogenic effect. Subsequently, microbubbles were observed to be capable of generating cavitation effect in ultrasonic fields, which could be further classified into stable and inertial cavitation: the former generated a microstreaming at the relatively lower ultrasound intensities, and the latter produced a shock wave and microjet in response to high-pressure fields (Hynynen et al., 2001, 2005; Pouliopoulos et al., 2020). Based on this characteristic, it was quickly realized that ultrasound combined with microbubble preparations may offer a suitable means of delivering small molecule drugs, genes, or other macromolecules into the brain by temporarily opening the tight junction of BBB (Unger et al., 2004; Sheikov et al., 2008). Over the years, various commercially available microbubble contrast agents have been launched successfully onto the market for clinical use (Hernot and Klibanov, 2008). Even so, there are also several unresolved questions about microbubbles, such as large particle size, poor stability, and short half-life in circulation (Konofagou, 2012). Remarkably, with the rise of nanotechnology, nanobubbles have shown great advantages in terms of penetration, stability, and therapeutic efficacy compared with traditional microbubbles and have become an increasingly popular research focus (Schroeder et al., 2010; Bing et al., 2018; Cheng et al., 2019).

#### Cluster 2: Management of Intracranial Tumors

At present, chemotherapy is still one of the most important therapeutic approaches for intracranial tumors, such as glioma and glioblastoma, but the special structure of BBB and blood-tumor barrier hinders most chemotherapeutic agents penetrating into tumor tissue (Beccaria et al., 2020). However, mounting preclinical evidence suggest that FUS have already shown great ability in improving delivery of multiple chemotherapeutic drugs, including temozolomide (Liu et al., 2014), liposomal doxorubicin (Yang et al., 2012; Escoffre et al., 2013), and carmustine (Liu et al., 2010b) and other oncotherapeutic molecules, such as nanoparticles (Curley et al., 2020), natural killer cells (Alkins et al., 2013), and monoclonal antibodies (Park et al., 2012), across the BBB. As far as we know, several clinical trials are also in progress by using ultrasound-induced BBB opening technique in brain tumor patients (Alkins et al., 2016; Idbaih et al., 2019; Beccaria et al., 2020). Taking one of the completed clinical trials as an example, Mainprize et al. (2019) performed MR-guided focused ultrasound combined with chemotherapeutic agents (liposomal doxorubicin or temozolomide) in five patients with newly diagnosed high-grade glioma at 1 day prior to surgical resection. Results of this study demonstrated that the whole procedure was well-tolerated without related adverse clinical or radiologic events. In addition, the drug concentration especially temozolomide in the sonicated tissue (reported in two cases) was increased obviously (Mainprize et al., 2019). Therefore, ultrasound-induced BBB opening is a promising adjunctive modality in enhancing the therapeutic efficacy of intracranial tumors. We will also continue to pay attention to the result of other clinical trials in the future.

#### Cluster 3: Study of Ultrasound Parameters and Mechanisms

Ultrasound is a propagating pressure wave with a frequency above the upper limit of human hearing (>20 kHz), which has the ability to transfer mechanical energy into biological tissues as a clinical diagnosis and treatment tool (Deckers and Moonen, 2010). At earlier stages, neurological ultrasound imaging was hindered due to its poor ability to focus and transmit ultrasound energy through the skull to the brain targets, as well as a lack of effective technologies for assessing the impact of ultrasound on nervous tissue. However, these limitations have been addressed with the technological developments. The appearance of variable electronic timing of transducer arrays and physically concave transducers allows focusing of ultrasound through the skulls of primates and humans (Qin et al., 2009). Likewise, MRI is capable of providing a detailed view of brain structure and evaluating the effects of ultrasound on brain tissue (Hynynen et al., 2001). From the safety point of view, the most important is that this technique does not cause progressive or irreversible damage to cells and tissues in the brain. Currently available studies have suggested that multiple ultrasound parameters including acoustic pressure, pulse length, frequency of the transducer, and pulse repetition frequency have shown to affect the reversibility of BBB permeability (Baseri et al., 2010; Leinenga et al., 2016; Snipstad et al., 2018). After setting the proper ultrasound parameters, repeated opening of the BBB is safe without causing overt damage according to the experimental findings from larger animals (McDannold et al., 2007; Dauba et al., 2020). However, it still remains uncertain whether long-term BBB opening is associated with a certain degree of tissue damage. Also, how the different types of mechanical effects lead to BBB opening and how to optimize the role of microbubbles have not yet been thoroughly studied (Cao et al., 2018; Krishna et al., 2018). Therefore, further research is still required to fully understand the influence of these parameters.

#### Cluster 4: Treatment of Neurodegenerative Diseases

Neurodegenerative diseases, like Alzheimer's disease, Parkinson's disease, multiple sclerosis, and others, are becoming increasingly prevalent as the global population ages. Although remarkable progress has been made over the past decade, there are currently few effective treatments and no cures available (Hung et al., 2010). Hitherto, the available therapies for these diseases are merely palliative, such as acetylcholinesterase inhibitors for Alzheimer's disease and levodopa and dopamine agonists for Parkinson's disease. However, just as the difficulties faced by oncotherapeutic agents, the BBB also inhibits their delivery to the degenerating brain. In principle, ultrasound-induced BBB opening technique can be combined and applied in the case of any CNS diseases. For instance, ultrasound, especially FUS, have a place in the symptomatic treatment of Alzheimer's disease, transiently opening the BBB to allow an unexpected entry of immunoglobulins and albumin (D'Haese et al., 2020; Dubey et al., 2020). Microglia activation, significant reduction in the amyloid plaque, and improvement in cognition were observed following this approach in preclinical models (Jordão et al., 2013; Leinenga and Götz, 2015) and clinical trial (Lipsman et al., 2018; Rezai et al., 2020). Of note, no matter with or without therapeutic agents, ultrasound seems to be capable of preventing the accumulation of misfolded and aggregated proteins, or removing them once formed, and the mechanisms involved remained unclear (Miller and O'Callaghan, 2017). Moreover, in addition to pharmacological therapies, in recent years, immunotherapy (Schwab et al., 2020), gene therapy (Fan et al., 2017), and stem cell therapy (De-Gioia et al., 2020) have also emerged as promising therapeutic modalities for neurodegenerative diseases; ultrasound-induced BBB opening also provides an exciting opportunity for these therapeutic molecules into a specific brain region. Of course, wider transition of this technique to clinical application will require more preclinical and clinical research prior to using it routinely.

#### Transition of Research Focus

Figure 9B summarizes the keyword occurrences with a time zone perspective according to their AAY. As is evident from this figure, early research prior to 2015, “microbubbles study” and “ultrasound parameters and mechanisms study” were the main concerns for researchers in the field. By contrast, keywords in cluster 2 and cluster 4 had the larger AAY compared with other clusters. This indicates that current research hotspot in the field has shifted to “management of intracranial tumors” (Alkins et al., 2016; Idbaih et al., 2019; Beccaria et al., 2020) and “treatment of neurodegenerative diseases” (Lipsman et al., 2018; D'Haese et al., 2020; Rezai et al., 2020). This result is in line with the development law of biomedical disciplines, that is, the ultimate goal of the exploration of basic research is to be transformed into clinical applications. As this technology gains recognition, it is expected to become one of the most important techniques for the treatment of CNS diseases in the near future.

### Burst Keywords as Indicators of Research Frontiers

Burst detection was used to identify keywords that have attracted the attention of peer scholars within a period of time. We detected significant burst keywords between 1998 through 2020 based on analysis of the 1,201 papers by CiteSpace. Among them, of specific interest were those keywords with research implications, which could indicate the evolution trend and predict research frontiers of ultrasound-induced BBB opening in the future. As we can see from the overall change tendency of burst keywords, the research hotspot has experienced a transition from basic research to clinical applications. Most notably, the burst of these six keywords including “Parkinson's disease” (Fan et al., 2017), “doxorubicin” (Kinoshita et al., 2006; Yang et al., 2012), “gold nanoparticle” (Sultan et al., 2018; Ye D. et al., 2018), “glioblastoma” (Idbaih et al., 2019), “gene therapy” (Timbie et al., 2015; Fan et al., 2017), and “Alzheimer's disease” (D'Haese et al., 2020; Dubey et al., 2020) is still ongoing, which have the most potential to continue to be the research hotspots and frontiers in the near future. Take gold nanoparticle as an example, among various inorganic and organic nanoparticles, gold nanoparticles possess many advantages, such as good biological compatibility, straightforward synthesis, and unique optical property and surface plasmon resonance (Boisselier and Astruc, 2009). These attributes could be exploited, either as drug carriers, or as active compounds in thermotherapy or photodynamic therapy, underlying the therapeutic applications. Multiple studies have confirmed that FUS in combination with microbubbles to induce BBB opening offered a promising strategy to focally enhance the delivery of gold nanoparticles or gold nanoclusters with therapeutic potential into the brain to improve the efficacy of tumor therapy (Coluccia et al., 2018; Sultan et al., 2018; Ohta et al., 2020). Therefore, the technique of ultrasound-induced BBB opening promises to expand the application of gold nanoparticles in neurological diseases.

## Limitations

There are some limitations applied for this study which need to be noted and addressed. (i) First, since our study is based on a single database from WoSCC, an implication is that we might have missed some relevant publications from other databases. Nevertheless, different databases have different ways to count the frequency of citations, thus it is not appropriate to merge various data from different databases, and most of the previous scientometric studies were conducted only by one single database (Teles et al., 2018; Yeung et al., 2018; Wang et al., 2020), and the reasons why we chose WoSCC have been described in detail previously (**Data Source**). (ii) Second, our study is focusing only on English literature, which leads to a certain deviation in the results of this study. As far as we have observed, for most non-English articles, the requirement cannot be met due to lack of English abstract or references (Chen et al., 2021). (iii) In terms of research methods, we only used classical methods, such as co-authorship, co-citation, and co-occurrence analysis, to analyze the retrieved literature. There are many other methods including bibliographic coupling analysis, cluster analysis from timezone view to conduct such studies.

## Conclusion

This study was undertaken to systematically summarize the knowledge structure and research frontiers regarding ultrasound-induced BBB opening by means of scientometric and visualization analysis, providing state-of-the-art and future perspectives. The results of the current study clearly demonstrate that there has been a surge of interest in research related to ultrasound induced BBB opening in recent years. The USA, and China made major contributions in this field, and the former was the country with the most productive publications, the highest H-index, the most funding. *Ultrasound in Medicine and Biology*, and *Journal of Controlled Release* were the most prominent journals. Radiology/Nuclear medicine/Medical imaging, Pharmacology/Pharmacy, and Neurosciences/Neurology can be considered the subject categories received the most attention. Hynynen K and Mcdannold N were key researchers with considerable academic influence. According to the occurrence analysis of keywords, four main research directions were identified: cluster 1 (microbubbles study), cluster 2 (management of intracranial tumors), cluster 3 (ultrasound parameters and mechanisms study), and cluster 4 (treatment of neurodegenerative diseases). Combining the analysis of keywords occurrence and burst, the current research hotspot has shifted from the basic research of ultrasound and microbubbles to management of intracranial tumors and neurodegenerative diseases. As a perspective future, Parkinson's disease, doxorubicin, gold nanoparticle, glioblastoma, gene therapy, and Alzheimer's disease may continue to be the research frontiers. In summary, our study provides a comprehensive and up-to-date overview in the field of ultrasound induced BBB opening from a global perspective. The results of the study could be interesting to scholars who have just entered the field to a better understanding of the current state, and policy makers in countries mentioned to improve risk assessments of funding investment.

## Data Availability Statement

The original contributions presented in the study are included in the article/Supplementary Material, further inquiries can be directed to the corresponding authors.

## Author Contributions

HW, YZ, ZS, and HY designed the study. HW, YZ, LT, and YW collected the data. HW, LX, BL, HY, and ZS analyzed the data and drafted the manuscript. ZS and HY revised and approved the final version of the manuscript. All authors have read and approved the submitted version.

## Conflict of Interest

The authors declare that the research was conducted in the absence of any commercial or financial relationships that could be construed as a potential conflict of interest.
