# Supplementary material for: Mapping Knowledge Structure and Research Frontiers of Ultrasound-Induced Blood-Brain Barrier Opening: A Scientometric Study
Source: Front Neurosci. 2021 Jul 14;15:706105. doi: 10.3389/fnins.2021.706105 (PMC8316975; doi:10.3389/fnins.2021.706105)
Supplement: Supplementary file 1 [file Table_1.docx]

Supplementary Material

# Supplementary Figures and Tables

## Supplementary Figure

**Supplementary Figure 1.** The top 15 research areas covered by the leading journals, and the total number of publications of each research area.


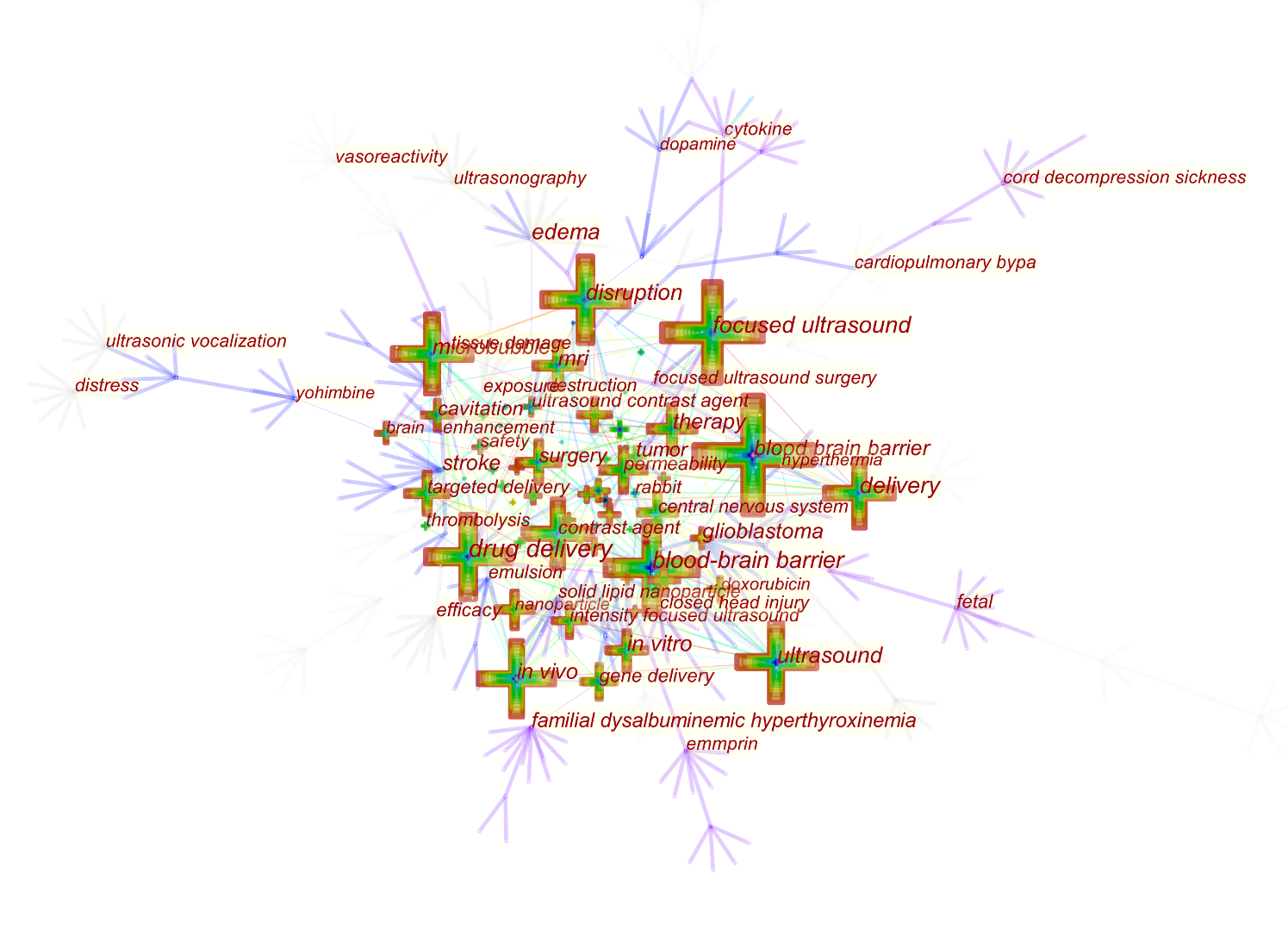


**Supplementary Figure 2.** A visualization knowledge map of high-frequency keywords generated by Citespace.
